# The Bioassay-Guided Isolation of Growth Inhibitors of Adult T-Cell Leukemia (ATL), from the Jamaican Plant *Hyptis verticillata*, and NMR Characterization of Hyptoside

**DOI:** 10.3390/molecules17089931

**Published:** 2012-08-17

**Authors:** Toshiyuki Hamada, Yohann White, Mitsuyoshi Nakashima, Yusuke Oiso, Masaki J. Fujita, Hiroaki Okamura, Tetsuo Iwagawa, Naomichi Arima

**Affiliations:** 1 Graduate School of Science and Engineering, Kagoshima University, 1-21-35 Korimoto, Kagoshima 890-0065, Japan; 2 Division of Hematology and Immunology, Center for Chronic Viral Diseases, Graduate School of Medical and Dental Sciences, Kagoshima University, 8-35-1 Sakuragaoka, Kagoshima 890-8544, Japan; 3 Priority Organization for Innovation and Excellence, Kumamoto University, 5-1 Oe-Honmachi, Kumamoto 860-8555, Japan

**Keywords:** ATL, bioassay-guided isolation, S1T, growth inhibitor, lignan, NMR, structure

## Abstract

Through bioassay-guided isolation, five compounds with growth inhibitory activity on S1T, an adult T-cell leukemia (ATL) cell line, were isolated from the crude methanol extract of the aerial parts of *Hyptis verticillata*.

## 1. Introduction

Adult T-cell leukemia (ATL) is an aggressive malignant disease caused by the human T-cell lymphotropic virus type I (HTLV-I). After infection with HTLV-I, 2–5% of carriers may develop ATL after a long latency period (30–50 years) [1]. The affected patients are frequently found in areas where HTLV-I infection is endemic, such as Japan, South America, the Caribbean Basin, West-Central Africa, Northern Iran, Southern India and other isolated tropical regions [2]. ATL has a poor prognosis, with a median survival of 13 months for the most aggressive form, the acute sub-type [3], being refractory to currently available combination chemotherapy. Therefore, the development of novel therapeutic methods to prevent the development of ATL or to prolong survival after its occurrence is warranted.

Traditionally, preparations from aerial parts of the plant *Hyptis verticillata* (Lamilaceae) have been used in Central America and the Caribbean for the treatment of gastrointestinal disorders [4] and skin infections [5]. Several compounds have been isolated from *H. verticillata*, including lignans [6,7], terpenes [8,9], and essential oils [10], but with relatively little focus on the anti-tumor effects of these compounds [6].

In the present study, the *in vitro* inhibitory effects on proliferation and the induction of apoptosis in S1T leukemia cells by the methanol extract of the dried aerial parts of the Jamaican *H. verticillata* are demonstrated. Also described are the bioassay-guided isolation, the structural elucidation and inhibitory activity against S1T cells of five lignans: hyptoside (**1**) [11], hyptinin (**2**) [7], *β*-peltatin (**3**) [12], 4′-demethyldesoxypodophyllotoxin (**4**) [13], and deoxypicropodophyllin (**5**) [14]. This is the first bioassay-guided isolation of anti-ATL compounds from plant extracts. Moreover, the unambiguous NMR chemical shift assignment of compound **1** by two-dimensional NMR techniques and the comparison of the ^1^H- and ^13^C-NMR chemical shifts of **1** with those previously assigned for hyptinin (**2**) [7] are also described.

## 2. Results and Discussion

**Figure 1:**
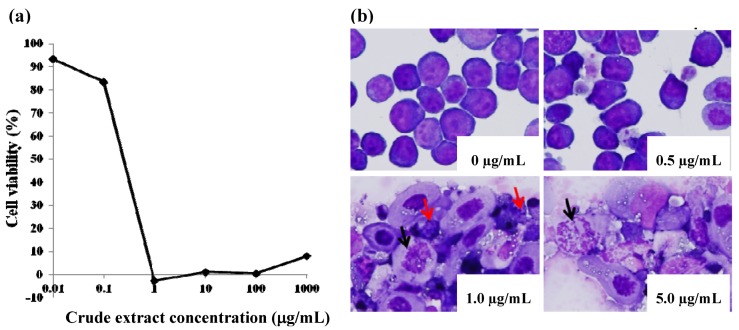
Cytotoxicity and morphologic assessment of apoptosis induction by *Hyptis verticillata* Jacq. crude extract. (**a**) Cytotoxicity against adult T-cell leukemia cell line, S1T; inhibitory concentration 50% (IC_50_) = 0.22 μg/mL. (**b**) May-Grünwald-Giemsa staining of S1T cells after incubation with the *Hyptis* extract at the dose indicated on each micrograph. Ten thousand cells were incubated alone or in the presence of increasing amounts of the extract. Disruption of nuclei (black arrows) and formation of apoptotic bodies (red arrows) are shown. Magnification ×400.

In the course of screening natural products for inhibitory effects against several human leukemia cell lines, the crude methanol extract of the aerial parts of *H. verticillata* showed significant cytotoxic effects against the oncoprotein-non-expressing HTLV-I-related leukemia cell line, S1T (Figure 1a), with an IC_50_ value of 0.22 μg/mL. As an indication of the relative toxicity or side effect potential of the compounds being investigated, cytotoxicity was also assayed using activated peripheral blood mononuclear cells (PBMCs) from normal healthy donors. The therapeutic index (ratio of cytotoxicity against healthy PBMCs to cytotoxicity against tumor cells) was approximately 100-fold for the *Hyptis* crude extract. To investigate whether cytotoxicity of the *Hyptis* crude extract was through programmed cell death (apoptosis), morphologic analyses were performed by May-Grünwald-Giemsa staining and microscopy. The *H. verticillata* crude extract resulted in the formation of apoptotic bodies and nuclear disruption in keeping with apoptosis induction in a dose-dependent manner (Figure 1b). 

**Figure 2:**
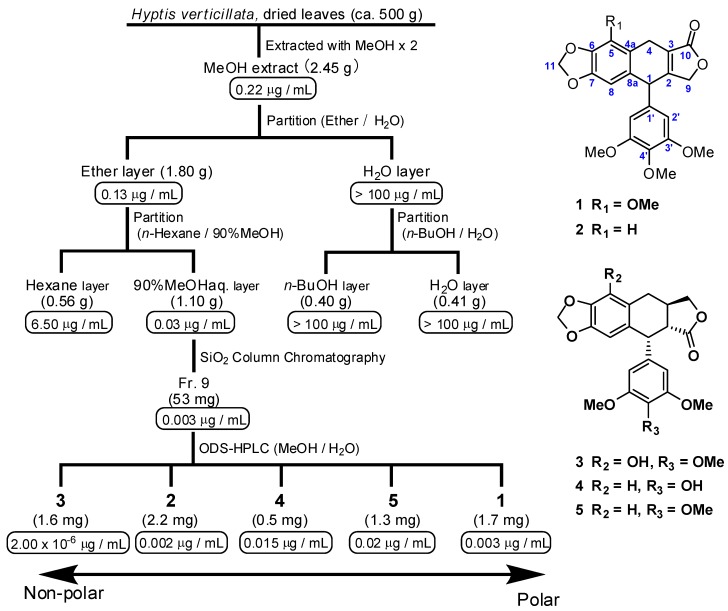
Bioassay-guided isolation scheme from dried leaves of *Hyptis verticillata* Jacq., and chemical structures of isolated, bioactive compounds.IC_50_ value (µg/mL) against the S1Tcell line is indicated below each of the respective extracts and fractions.

The principal components of the *H. verticillata* extract responsible for the anti-ATL effects were isolated (Figure 2). The MeOH extracts were partitioned between H_2_O and diethyl ether. The ether soluble portion (1.78 g) was further partitioned between 90% MeOH and *n-*hexane. The active 90% MeOH phase (1.07 g) was fractionated by silica gel flash chromatography, followed by ODS-HPLC to yield five ATL inhibitory lignans **1**–**5**.

Hyptoside (**1**) was obtained as a colorless amorphous solid. On the basis of HR-FAB mass spectrometry (pseudomolecular ion at *m/z* 449.1216 [M+Na]^+^) and the ^13^C-NMR and DEPT spectra of **1**, its molecular formula was established as C_23_H_22_O_8_, indicating eight degrees of unsaturation. The UV (275 nm) and IR data (1751 and 1589 cm^−1^) of **1** suggested a conjugated carbonyl system, while the IR peak at 2921 cm^−1^ indicated the presence of methoxy groups in **1**. The spectroscopic data, including ^1^H- and ^13^C-NMR (Table 1), were similar but not completely identical to those of hyptinin (**2**). An additional methoxy group at δ_H_ 4.07 showed an HMBC cross-peak with C-5, permitting the location of the methoxy group at C-5. Conclusive proof that the lactone carbonyl is bonded to C-3 was obtained by HMBC experiments: H-4 (δ_H_ 3.64) showed the HMBC correlations to C-10 (δ_C_ 172.2). On the basis of this information, the structure of **1** was identified as 5-methoxy hyptinin and its ^13^C-NMR data were assigned by the HMQC and HMBC correlations, as shown in Table 1. In addition, hyptinin (**2**) [7], *β*-peltatin (**3**) [12], 4′-demethyldesoxypodophyllotoxin (**4**) [13] and deoxypicropodophyllin (**5**) [14] were identified by comparison with data from the literature. The absolute stereochemistry of compounds **1** and **2** remains to be determined, as attempts at hydrogenation were unsuccessful due to the paucity of sample obtained; signs of optical rotation for these compounds, however, were not identical to those reported for hyptinin.

Anti-tumor effects of compounds **1**–**5** were investigated by WST-8 assay. All compounds showed significant cytotoxic effects against the HTLV-I-related leukemia cell line, S1T. The 50% inhibitory concentration (IC_50_) of each of compounds **1**–**5** was 7.0, 5.1, 4.8 × 10^−3^, 39 and 50 nM, respectively. This indicates that these lignan-related compounds showed strong inhibitory effects on cell proliferation, warranting further investigation with the aim of developing novel anti-ATL drugs. Some podophyllotoxin-like lignans, such as *β*-peltatin (**3**) and its synthetically derived etoposide, are well-known anticancer agents [15]; while the anti-tumor effects of hyptoside (**1**) and hyptinin (**2**), structurally different types of lignans, had prior been less well characterized.

In summary, the MeOH extract of Jamaican *H. verticillata* shows *in vitro* inhibitory effects on proliferation and apoptosis induction. It should be noted that the same plant species collected in Japan did not exhibit such cytotoxic effects (data not shown). A proliferation inhibition assay using S1T leukemia cells was used for the bioassay-guided isolation of active constituents from the crude plant extract, resulting in the identification of five bioactive lignans. Recently, hyptoside (**1**) has been shown to be cytotoxic against a variety of cancer cells, including several multidrug-resistant cancer cells [11]. Further studies on the biological effects of **1** are currently being conducted. It is expected that these additional studies for elucidating the mechanism of action, anti-tumor effects and toxicity profile of hyptoside in a recently developed ATL-mouse model will aid in the development of novel drugs for the treatment of ATL.

**Table 1:** NMR spectral data of **1** in CDCl_3_ at 300 K.

No.	δ_C_		δ_H_ (mult., *J* = Hz)	HMBC correlations
1	42.7		4.76 (m)	127.6 (C-3), 138.2 (C-1′), 105.5 (C-2′)
2	158.0			
3	127.6			
4	24.3		3.60 (dd, 22.5, 4.5)	158.0 (C-2), 127.6 (C-3), 130.5 (C-4a),
			3.64 (dd, 22.5, 4.1)	140.1 (C-5), 116.2 (C-8a), 172.2 (C-10)
4a	130.5			
5	140.1			
6	148.8			
7	134.5			
8	103.3		6.34 (s)	42.7 (C-1), 148.8 (C-6), 134.5 (C-7), 116.2 (C-8a)
8a	116.2			
9	71.1		4.76 (brd, 16.8)	158.0 (C-2), 127.6 (C-3)
			4.86 (d, 16.8)	158.0 (C-2), 127.6 (C-3), 172.2 (C-10)
10	172.2			
11	100.9		5.91 (d, 1.6)	148.4 (C-6), 134.5 (C-7)
			5.92 (d, 1.6)	148.4 (C-6), 134.5 (C-7)
1′	138.2			
2′ & 6′	105.5		6.34 (brs)	42.7 (C-1), 138.2 (C-1′), 105.5 (C-2′ or C-6′), 153.2 (C-3′ or C-5′), 137.9 (C-4′)
3′ & 5′	153.2			
4′	137.9			
5-OMe	59.3		4.07 (s)	140.1 (C-5)
3′-OMe & 5′-OMe	56.1		3.76 (s)	153.2 (C-3′ or C-5′)
4′-OMe	60.7		3.76 (s)	137.9 (C-4′)

## 3. Experimental

### 3.1. General Procedures

Optical rotation was measured at 25 °C on a JASCO DIP-370S polarimeter. NMR spectra were recorded with JEOL ECX400 and ECX600 spectrometers. UV and IR spectra were recorded on a UV-210 and a JASCO FT/IR 5300. FAB mass spectra were obtained using a JEOL JMX-SX/SX 102A spectrometer. Column chromatography was performed with silica gel 60 (Merck, 70–230 μm). Silica gel 60F plates (Merck, 0.25 mm thick) were used for TLC. HPLC was performed using a Waters 501 HPLC pump with a Shodex UV-41 detector. A C_18_ column (4.6 mm *ϕ* × 250 mm) was used for HPLC.

### 3.2. Biological Materials

The plant samples were collected several times by hand during the summer of 2007 in Kingston, Jamaica. Voucher specimens were deposited at the herbarium of the Department of Life Sciences, University of the West Indies, Mona, Jamaica.

### 3.3. Extraction and Isolation

The plant specimens (500 g, wet weight) were chopped into small pieces and extracted with MeOH (500 mL) twice for 1~2 weeks. Extracts were concentrated under reduced pressure at 40–45 °C to yield a dark green resin. The MeOH extract (4.65 g) was partitioned between H_2_O (1 L) and diethyl ether (3 L). The ether extract (1.78 g) was further partitioned between MeOH/H_2_O (9:1) and *n-*hexane. The aq MeOH layer (1.07 g) was subjected to silica gel flash chromatography (hexane/EtOAc = 8:2–1:1, gradient) to give 20 fractions. The fractions eluted with hexane/EtOAc (2:3) were collected and purified by ODS-HPLC on Cosmosil 5C_18_-AR with MeOH/H_2_O (1:1) to furnish compounds **1** (1.7 mg), **2** (2.2 mg), **3** (1.6 mg), **4** (0.5 mg) and **5** (1.3 mg).

### 3.4. Analytical Data

*Hyptoside* (**1**): colorless amorphous solid; [α]^25^_D_ −63.0 (*c* 0.05, CH_2_Cl_2_); UV λ_max_ (CH_2_Cl_2_) 331 (logε = 3.209), 275 (3.531); IR (film): 2929, 1751, 1581, 1459, 1235, 1125 cm^−1^: ^1^H and ^13^C-NMR (CDCl_3_) see Table 1. HRFABMS *m/z* 449.1216 [M+Na]^+^ (calcd for C_23_H_22_O_8_Na, +0.4 mmu).

*Hyptinin* (**2**): colorless amorphous solid; [α]^25^_D_ −157.5 (*c* 0.01, CH_2_Cl_2_); UV λ_max_ (CH_2_Cl_2_) 351 (2.969), 294 (3.591); IR (film): 2921, 1751, 1589, 1484, 1231, 1124 cm^−1^: ^1^H-NMR (400 MHz, CDCl_3_): δ 6.72 (1H, *s*), 6.63 (1H, *s*), 6.37 (2H, *s*), 5.95 (2H, br*s*), 4.87 (1H, *d*, *J* = 17), 4.81 (1H, *dd*, *J* = 17.2, 2.0), 4.81 (1H, *m*), 3.87 (1H, *dd*, *J* = 22.5, 4.5), 3.79 (3H, *s*), 3.78 (6H, *s*), 3.63 (1H, *dd*, *J* = 22.5, 4.0); FABMS *m/z* 497.2 [M+H]^+^.

*β-Peltatin* (**3**): colorless amorphous solid; [α]^25^_D_ −132.5 (*c* 0.05, CH_2_Cl_2_); UV λ_max_ (CH_2_Cl_2_) 274 (2.892); IR (film): 2935, 1764, 1588, 1463, 1248, 1124 cm^−1^: MS and NMR data identical to those reported [12]. 

*4′-Demethyldesoxypodophyllotoxin* (**4**): colorless amorphous solid; [α]^25^_D_ −89.3 (*c* 0.08, CH_2_Cl_2_); UV λ_max_ (CH_2_Cl_2_) 294 (0.202); IR (film): 2921, 1758, 1606, 1480, 1456, 1217 cm^−1^: MS and NMR data comparable to literature values [13].

*Deoxypicropodophyllin* (**5**): colorless amorphous solid; [α]^25^_D_ −130.7 (*c* 0.04, CH_2_Cl_2_); UV λ_max_ (CH_2_Cl_2_) 293 (3.340); IR (film): 2921, 1764, 1588, 1483, 1239, 1125 cm^−1^: MS and NMR data were identical to those reported for deoxypicropodophyllin [14]. 

### 3.5. Cell Lines and Cultures

The adult T-cell leukemia cell line S1T was maintained in RPMI-1640 supplemented with 10% fetal bovine serum, 100 U/mL penicillin, 100 μg/mL streptomycin, and 2 mM L-glutamate. Generally, cell cultures were split every 2 to 3 days, and used for *in vitro* assays during the log phase of growth.

### 3.6. Cytotoxicity

The cells were cultured at the density of 1 × 10^4^ cells per well in at least triplicates in the absence or presence of a test sample in ten-fold dilutions for 72 hours in flat bottom 96-well plates at 37 degrees celcius in a humidified water-jacketed CO_2_ incubator. The inhibition of cell proliferation was determined using a 2-(2-methoxy-4-nitrophenyl)-3-(4-nitrophenyl)-5-(2,4-disulfophenyl)-2*H*-tetrazol-ium monosodium salt (WST-8) assay kit (Dojindo, Kumamoto, Japan). The viable cells convert the WST-8 tetrazolium salt into a water-soluble formazan. The concentration at which cell proliferation is inhibited by 50% compared to untreated control cells is expressed as the IC_50_.

### 3.7. Morphologic Assessment of Apoptosis Induction

Apoptosis induction was assessed by incubating leukemia cell lines or ATL patient PBMCs for 48 hours in 24-well flat bottom plates (IWAKI, Japan) at a suspension of 2 × 10^4^ and 2 × 10^5^ cells per mL, respectively, in the presence or absence of test agents. Cells were then transferred to glass slides using a cytospin apparatus (Cytospin, Shandon, Amstoor, UK) followed by May-Grünwald Giemsa staining.

## 4. Conclusions

The crude methanol extract of the aerial parts of *H. verticillata* showed significant cytotoxic effects against S1T, an adult T-cell leukemia (ATL) cell line. Through several steps of *in vitro* based bioassay-guided fractionation, five growth inhibitory compounds were isolated from the extract. The structures of these compounds were identified as lignan-type metabolites by UV, FT-IR, MS, and 1-and 2-D NMR spectroscopy. These five compounds displayed high cytotoxicity against S1T.
